# Profile of sexually transmitted infections causing urethritis and a related inflammatory reaction in urine among heterosexual males: A flow-cytometry study

**DOI:** 10.1371/journal.pone.0242227

**Published:** 2020-12-02

**Authors:** Stanislav Tjagur, Reet Mändar, Margus Punab

**Affiliations:** 1 Andrology Centre, Tartu University Hospital, Tartu, Estonia; 2 Faculty of Medicine, University of Tartu, Tartu, Estonia; 3 Department of Microbiology, Institute of Biomedicine and Translational Medicine, Faculty of Medicine, University of Tartu, Tartu, Estonia; 4 Competence Centre on Health Technologies, Tartu, Estonia; 5 Institute of Clinical Medicine, Faculty of Medicine, University of Tartu, Tartu, Estonia; Xavier Bichat Medical School, INSERM-CNRS - Université Paris Diderot, FRANCE

## Abstract

**Background:**

Information about the use of flow cytometry in the diagnosis of male urethritis is scarce. The current study aims to evaluate the performance of flow cytometry on first-voided urine in males with infectious urethritis (*Chlamydia trachomatis*, *Neisseria gonorrhoeae*, *Mycoplasma genitalium* and *Trichomonas vaginalis*).

**Methods:**

Male patients of the Andrology Centre (Tartu University Hospital, Estonia) were recruited during the period March 2015 –January 2018. Cases included 306 patients with infectious urethritis caused by *Chlamydia trachomatis*, *Neisseria gonorrhoeae*, *Mycoplasma genitalium* and/or *Trichomonas vaginalis*. The control group consisted of 192 patients without uro-genital complaints, negative tests for *C*. *trachomatis*, *N*. *gonorrhoeae*, *M*. *genitalium* and *T*. *vaginalis* from first-voided urine and no inflammation in first-voided urine, mid-stream urine and urine after prostate massage. *C*. *trachomatis*, *N*. *gonorrhoeae*, *M*. *genitalium* and *T*. *vaginalis* were detected from first-voided urine using polymerase chain reaction (PCR) method. First-voided urine was analysed using urine particle analyzer Sysmex UF-500i.

**Results:**

The most prevalent infection was chlamydia (64.1%), followed by *Mycoplasma genitalium* (20.9%), gonorrhoea (7.8%) and trichomoniasis (1.6%). Gonorrhoea caused the highest flow-cytometric leucocyte/bacteria count, followed by chlamydia and *Mycoplasma genitalium*. *Trichomonas vaginalis* showed nearly absent inflammation in first-voided urine. Using an empiric flow-cytometry diagnostic threshold for urethritis in first-voided urine (leucocytes ≥ 15/μl and bacteria ≥ 20/μl) the total calculated sensitivity was over 90%. However, when applying such criteria for deciding whether to perform first-voided urine PCR for *C*. *trachomatis*, *N*. *gonorrhoeae*, *M*. *genitalium* and *T*. *vaginalis* or not, we could miss 23 cases with infectious urethritis that makes up 7,5% of all proven cases.

**Conclusions:**

Flow cytometry of first-voided urine can be considered as a rapid and objective screening method in case of suspected male infectious urethritis.

## Introduction

Sexually transmitted infections (STI) affect the health of people worldwide causing several adverse consequences. According to the World Health Organization, the global prevalence estimates of STIs for men in 2016 were 2.7% for chlamydia, 0.7% for gonorrhoea and 0.6% for trichomoniasis. These numbers remain generally unchanged in comparison with the global data for 2012 [1].

According to the European guidelines, diagnosis of non-gonococcal urethritis should be confirmed by demonstrating polymorphonuclear leukocytes from the anterior urethra using a Gram-stained (GSS) or methylene blue-stained urethral smear [2]. However, a marked degree of both intra-observer and inter-observer variation has been found in the interpretation of the urethral GSS [3, 4]. At the same time, in recent years, new technologies have emerged in the field of urinalysis methodology, offering new quick and standardized opportunities in everyday clinical practice. One of such technologies is urine particle flow cytometry that improves urine particles’ count precision and accuracy compared with conventional visual microscopy and offers significant labor reduction [5]. Compared with urethral swabbing, this technique is simple to perform, automated, fast (results within a few minutes), offers high sample throughput and is not invasive. However, the majority of studies have controlled the performance of flow cytometry for urinary tract infections using midstream urine [6], and there is only limited information about the performance and cut-off values for flow cytometry in diagnosis of STIs associated with urethritis [7]. In a previous study we highlighted the symptomatology of STIs associated with urethritis and could not provide a detailed description of inflammatory reaction for each particular pathogen due to the shortcomings associated with urine dipstick analysis [8]. This time we have aimed to find the optimal cut-off levels for triggering diagnostics of STIs associated with urethritis with the aim of improving the cost-effectiveness of the management of infectious urethritis for heterosexual men in a busy outpatient clinic.

We aimed to evaluate the performance of flow cytometry on first-voided urine in patients with infectious urethritis (*Chlamydia trachomatis* [CT], *Neisseria gonorrhoeae* [NG], *Mycoplasma genitalium* [MG] and *Trichomonas vaginalis* [TV]) compared to healthy control group consulting the andrologist at the Andrology Centre of Tartu University Hospital in Estonia.

## Methods

### Study subjects

The study was organized prospectively. Patients of the Andrology Centre of Tartu University Hospital in Estonia were examined during the period from March 2015 to January 2018. A group of cases and a control group were recruited for the study. The age of the participants ranged from 18 to 50 years in both groups.

**The group of cases** consisted of patients who referred or self-referred (i.e., paid for the services by themselves) to the Andrology Centre either for a STI control after a case of high-risk sexual behavior (i.e. a sex with prostitute with/without protection; an oral sex with a prostitute without protection; a one-night stand sex with/without protection; oral sex without protection with a random partner; more than one regular sexual partner during the last six months), for fertility check, or for prophylactic health control. The recruitment criteria for the cases were the following: 1) The patients had done all the four STI PCR tests from first voided urine for CT, NG, TV and MG; 2) Only heterosexual patients were included; 3) Patients admitted for STI control after STI treatment were excluded. In total, 310 cases with positive STIs were identified. Amongst them, four STI positive subjects were excluded for the following reasons: in three cases first voided urine flow cytometry had not been performed and in one case the STI tests had been performed from a urethral smear. The final group of cases consisted of 306 STI-positive male patients. Among them, there was no information about the volume of the first voided urine in case of 87 individuals. Asymptomatic patients were also included in the group of cases. The clearly stated symptomatic/asymptomatic status of the patients in the group of cases could be retrieved from their medical history for 216 patients; among them, 53 patients (24.5%) were asymptomatic and 163 patients (75.5%) had complaints (a discharge from the urethra, redness of the urethral opening, persistent discomfort in the urethra, persistent or frequently recurring inflammation of the prepuce).

For **the control group**, we defined uniquely strict inclusion criteria and they were recruited from subjects who visited the Andrology Centre either for prophylactic purposes or for fertility check. The recruitment criteria for the control group were as following: 1) Subjects did not present any symptoms suspicious for uro-genital infections or inflammations (a discharge from the urethra, redness of the urethral opening, persistent discomfort in the urethra, persistent or frequently recurring inflammation of the prepuce); 2) Patients who had performed first-voided urine, mid-stream urine and urine after prostate massage with no inflammation found in any of these samples. Our empirical cut-off values for inflammation using flow-cytometry were as following: for first-voided urine and midstream urine—leucocytes ≥ 15/μl, bacteria ≥ 20/μl; for urine after prostate massage—leucocytes ≥ 40/μl, bacteria ≥100/μl; 3) The time frame between providing of first-voided and midstream urine/urine after the prostate massage was ≤ 14 days (median 7 days); 4) Patients had done all four STI PCR tests from the first voided urine for CT, NG, TV and MG; 5) Only heterosexual patients were included. One hundred and ninety two (192) patients were identified as the control group. All the patients in the control group had a registered volume of first-voided urine. Among the 192 patients of the control group, 143 patients also had performed semen analysis according to the WHO recommendations [9] and there was no inflammation in the semen analyses (concentration of white blood cells was below 1 million/ml).

The cutoff values for the bacteria and leucocyte count in first-voided urine, midstream urine and urine after prostate massage were derived from the patients’ cohort of the Andrology Centre of Tartu University Hospital. A complex set of methods was used—The International Prostate Symptom Score (IPSS); the National Institutes of Health Chronic Prostatitis Symptom Index (NIH-CPSI); performing digital rectal examination and determining signs of uro-genital infections or inflammations (a discharge from urethra, redness of the urethral opening, persistent discomfort in the urethra, persistent or frequently recurring inflammation of the prepuce).

Each patient of the control and cases’ group was screened for CT, NG, TV and MG from first-voided urine and had a flow-cytometric analysis of first-voided urine. A flow-cytometric analysis of midstream and post-massage urine was performed only in the patients of the control group. The reason for eliminating STI pathogen-negative patients with inflammation in first-voided and/or mid-stream urine from the control group and the cases’ group was the possible risk of including patients with chronic bacterial prostatitis (NIH2 group) [10, 11] or other etiological factors of urethritis (i. e. other bacterial pathogens, viral pathogens, mechanical factors) [12, 13] which could bias the flow-cytometric counts of bacteria and leucocytes in first-voided urine.

### Ethics statement

Participation in the study was voluntary. The study was approved by the Ethics Review Committee on Human Research of the University of Tartu, Estonia, permission 228/M-32 (26.08.2013), amended with 254/M-17 (21.12.2015). The study was conducted according to the Declaration of Helsinki principles. All participants provided written informed consent.

### Clinical exam

Each patient of the cases’ group passed a standard physical examination consisting of external genital examination; digital rectal examination was performed if necessary. Each patient of the control group passed standard physical examination consisting of external genital examination and digital rectal examination.

### Sample collection

First-voided urine was defined as first morning urine or urine provided at least 2–3 hours after last urination. The first-voided urine was performed at the Andrology Centre or brought by the patient from home. The first-voided urine was collected into a pre-weighted aseptic 150 ml urine container (Kartell S.p.A., Italy) for polymerase chain reaction (PCR) studies and for urine flow cytometry. In cases when the patient decided to bring first-voided urine from home a pre-weighted aseptic 150 ml urine container (Kartell S.p.A., Italy) was given to the patient by the clinic staff and the patient was instructed to bring the sample to the Andrology Centre within 1 hour after collection if at room temperature (+ 24°C), or within four hours if the sample was stored at + 4°C. Each patient was asked to provide 15–20 ml of first-voided urine. The midstream and post-massage urine were always collected at the Andrology Centre on the same day. The midstream urine was collected either right after first-voided urine collection or after providing 15–20 ml of urine if the test was planned on a separate day. Patients were told to collect 50–80 ml of the midstream urine. The post-massage urine was collected after a prostate massage that lasted for 2–3 minutes—the prostate secretion and the post-massage urine were collected into the same urine container with the purpose of saving the patient’s time and avoiding mixing up different collection tubes by the patient. The obligatory prerequisite before the massage of the prostate was sexual abstinence for 3–7 days before the procedure. The flow-cytometric analysis of urine samples was performed within one hour after sample’s arrival at the Andrology Centre. In case the flow-cytometric analysis of the urine sample could not be performed on the same day, the urine sample was stored in a refrigerator (+4°C) at the Andrology Centre and the analysis was performed on the following day.

### Urine analysis

The analysis of urine specimens was done by an experienced lab technician.

The weight of the first-voided urine was calculated extracting the weight of the pre-weighted urine container. Each urine container was weighted separately using electronic scales. The weight of 1 gram of first-voided urine was approximated with the volume of 1 milliliter of first-voided urine.

The concentration and total count of white blood cells and bacteria in urine were analyzed using urine flow cytometry. The analyses were performed using fully automated urine particle analyzer Sysmex UF-500i (manufactured by Sysmex Corporation, 1-5-1 Wakinohama-Kaigandori, Chuo-ku, Kobe 651–0073, Japan) according to the producer’s instructions at the Andrology Centre of Tartu University Hospital. A detailed description of the working principles of the UF-500i machine is presented in S1 Table. Attention is required in case of flow-cytometric detection of bacteria count in first-voided urine regarding CT, MG and TV positive cases, as CT is obligatorily intracellular and MG is a facultatively intracellular pathogen and flow-cytometer cannot detect intracellular bacteria, while TV is a non-bacterial agent. It is important to keep in mind that flow-cytometric dyes used in the analyzer Sysmex UF-500i and UF-1000i do not distinguish bacterial species and thus the flow cytometer only describes the presence or absence of bacteria in the urine sample. Sysmex has developed additional software for their urine flow cytometers that can give additional information on bacterial morphology, which may guide the physician in the choice of antibacterial treatment, but the utility of this software has been questioned in the study by Geerts et al. [14].

Using the measured concentrations of leucocytes and bacteria in the urine sample by flow cytometry, the total counts of white blood cells and bacteria were calculated, too, using the following formulas:

$$total\;count\;of\;leucocytes\;=\;[volume\;of\;first-voided\;urine\;sample,\;in\;grams]\;\times \;[concentration\;of\;leucocytes\;in\;first-voided\;urine\;sample,\;{\mu l}^{-1}]\;\times 1000$$

$$total\;count\;of\;bacteria\;=\;[volume\;of\;first-voided\;urine\;sample,\;in\;grams]\;\times \;[concentration\;of\;bacteria\;in\;first-\;voided\;urine\;sample,\;{\mu l}^{-1}]\;\times 1000$$

### Detection of urethritis-associated sexually transmitted infections

Urethritis-associated STIs were detected from the first-voided urine using a PCR method (*C*. *trachomatis* and *N*. *gonorrhoeae* DNA by Roche/ cobas^®^ 4800 CT/NG Test; *M*. *genitalium* DNA by Sacace Biotehnologies/Mycoplasma genitalium Real-TM; *T*. *vaginalis* DNA by Sacace Biotehnologies/Trichomonas vaginalis Real-TM) according to the manufacturer’s instructions, at the United Laboratory of Tartu University Hospital. All STI-positive patients were contacted for appropriate treatment and follow-up. They were told to inform and motivate sexual contacts to complete appropriate analyses and treatment.

### Statistical analysis

Statistical analyses were performed using Microsoft Excel software (Microsoft Corporation) and RStudio software (RStudio Inc.). Except age, the data about first-voided urine volume, total count and concentration of leucocytes and bacteria was with non-parametric statistical distribution. For comparison of the basic parameters of the control and cases’ groups a non-parametric Wilcoxon test was used. P-value < 0.05 was accepted as a statistically significant difference. Spearman correlation was used to assess the correlation between the concentration of leucocytes and bacteria. To assess the difference between the leucocyte and bacteria concentration/total count in different groups of STI-positive patients with monoinfection or combined infection Kruskal-Wallis test was used. Mann-Whitney test with Bonferroni correction (using 10 tests, corrected P-value < 0.005; using 3 tests, corrected P-value < 0.017) was used to assess the statistical significance of the difference of concentration and the total count of bacteria and leucocytes between different patients’ groups with STI monoinfection and combined infections.

## Results

The profile of the different STI agents and their combinations is shown in Table 1. The most prevalent monoinfection was CT (64.1%), followed by MG (20.9%) and NG (7.8%) defined by a PCR method. The prevalence of TV was low (1.6%). The total proportion of different combined infections was 5.6%. Because of the proportion of combined infections was low, the following data describe only STI monoinfections, while the information about combined infections is provided in S2 and S3 Tables.

**Table 1:** Profile of urethritis-associated sexually transmitted infections (STI) defined by a polymerase chain reaction (PCR) method among the male patients consulting an andrologist after high-risk sexual behavior or for prophylactic control or fertility check.

STI agent	Profile of urethritis-associated STIs in cases’ group (N = 306)	
	N	%
**Monoinfections**	**289**	**94,4**
*Chlamydia trachomatis*	196	64,1
*Mycoplasma genitalium*	64	20,9
*Neisseria gonorrhoeae*	24	7,8
*Trichomonas vaginalis*	5	1,6
**Combinations**	**17**	**5,6**
CT + NG	11	3,6
CT + MG	4	1,3
MG + NG	1	0,3
CT + NG + MG	1	0,3

Abbreviations: CT, *Chlamydia trachomatis*; NG, *Neisseria gonorrhoeae*; MG, *Mycoplasma genitalium*; TV, *Trichomonas vaginalis*.

**Fig 1:**
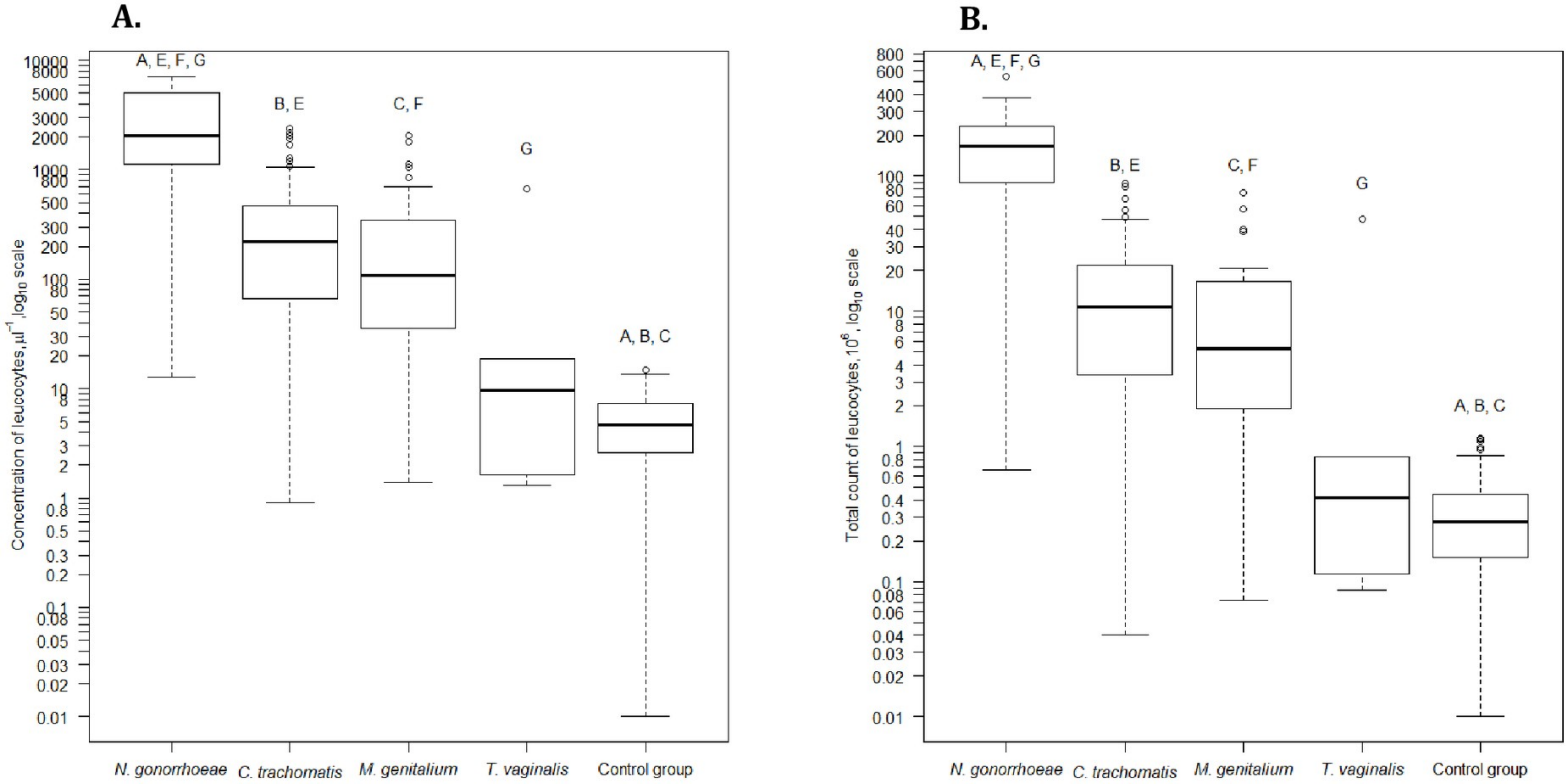
The distribution of leucocytes in first-voided urine according to flow cytometry among urethritis-associated STI-positive patients according to each STI pathogen, by concentration of leucocytes (A) and total count of leucocytes (B). Concentration of leucocytes per 1 μl of first-voided urine. Only STI monoinfections are displayed, 17 cases with combined STI have been excluded. For Fig 1A patients with and without sample volume were included. For Fig 1B only patients with sample volume were included (excluding 81 patients with monoinfectious STI: 55 cases with *C*. *trachomatis*, 19 cases with *M*. *genitalium*, 7 cases with *N*. *gonorrhoeae*). Mann-Whitney test with Bonferroni correction for 10 tests [A: Control *vs*. *N*. *gonorrhoeae*, B: Control *vs*. *C*. *trachomatis*, C: Control *vs*. *M*. *genitalium*, D: Control *vs*. *T*. *vaginalis*, E: *N*. *gonorrhoeae vs*. *C*. *trachomatis*, F: *N*. *gonorrhoeae vs*. *M*. *genitalium*, G: *N*. *gonorrhoeae vs*. *T*. *vaginalis*, H: *C*. *trachomatis vs*. *M*. *genitalium*, I: *C*. *trachomatis vs*. *T*. *vaginalis*, J: *M*. *genitalium vs*. *T*. *vaginalis*]. A, B, C, E, F, G—p<0.005.

**Fig 2:**
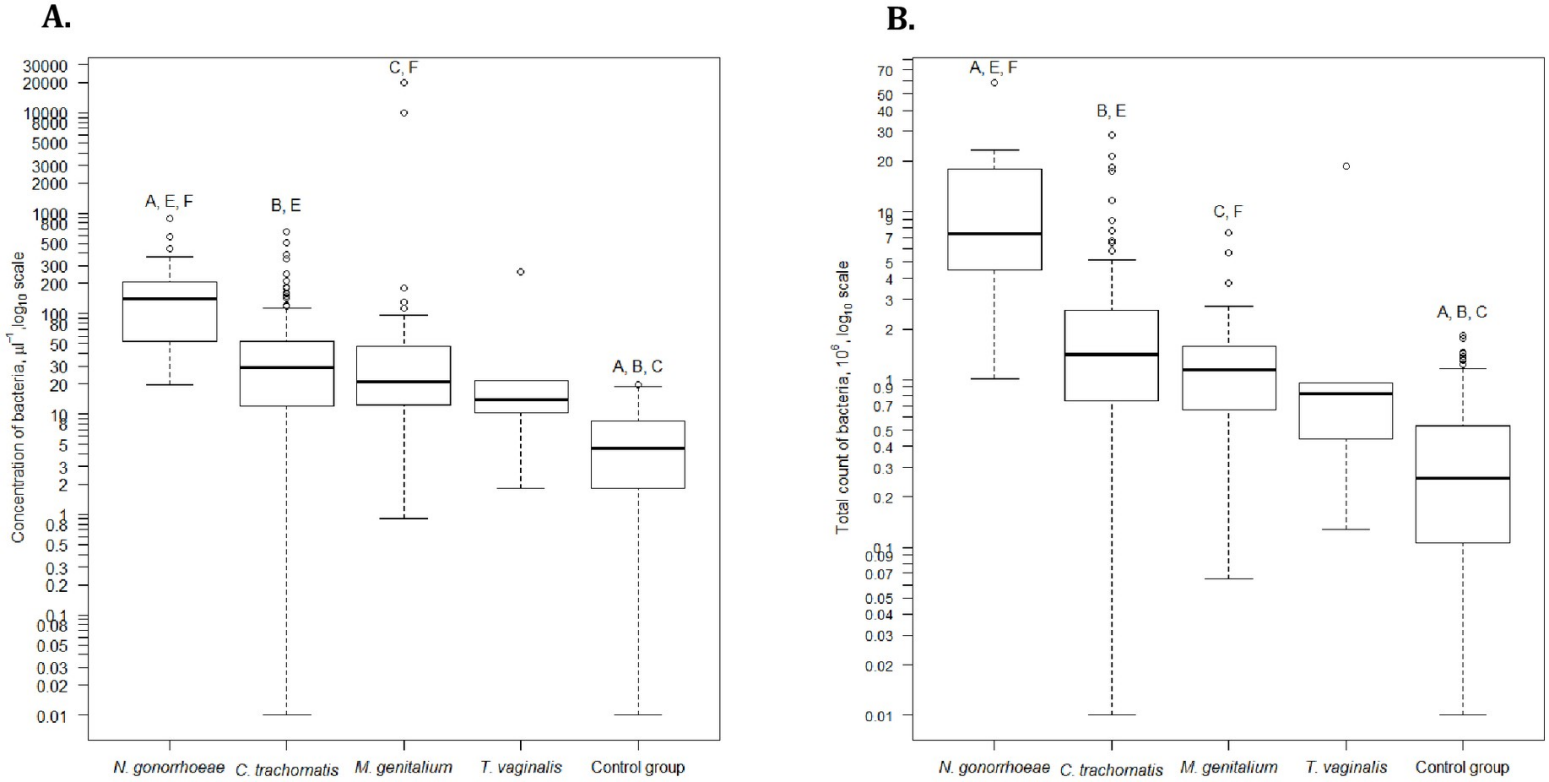
The distribution of bacteria in first-voided urine according to flow cytometry in case of each urethritis-associated STI pathogen. Concentration of bacteria (A) and total count of bacteria (B). Concentration of bacteria per 1 μl of first-voided urine. Only STI monoinfections are displayed, 17 cases with combined STI have been excluded. For Fig 1A patients with and without sample volume were included. For Fig 1B only patients with sample volume were included (excluding 81 patients with monoinfectious STI: 55 cases with *C*. *trachomatis*, 19 cases with *M*. *genitalium*, 7 cases with *N*. *gonorrhoeae*). Mann-Whitney test with Bonferroni correction for 10 tests [A: Control *vs*. *N*. *gonorrhoeae*, B: Control *vs*. *C*. *trachomatis*, C: Control *vs*. *M*. *genitalium*, D: Control *vs*. *T*. *vaginalis*, E: *N*. *gonorrhoeae vs*. *C*. *trachomatis*, F: *N*. *gonorrhoeae vs*. *M*. *genitalium*, G: *N*. *gonorrhoeae vs*. *T*. *vaginalis*, H: *C*. *trachomatis vs*. *M*. *genitalium*, I: *C*. *trachomatis vs*. *T*. *vaginalis*, J: *M*. *genitalium vs*. *T*. *vaginalis*]. A, B, C, E, F—p<0.005.

The total flow-cytometric counts and concentrations of bacteria and leukocytes in the first-voided urine were significantly different between the patients with and without STI (Table 2). The distribution of leucocytes in first-voided urine according to each pathogen is shown in Fig 1. NG caused the highest concentration and total count of leucocytes; TV showed nearly absent inflammatory reaction in first-voided urine; while CT and MG showed moderate inflammation. We also analyzed the flow-cytometric counts and concentrations of bacteria in case of each pathogen (Fig 2). Similarly to the inflammatory reaction, both parameters were the highest in case of NG and lowest in case of TV. CT and MG displayed quite similar moderate values.

**Table 2:** Basic parameters of study subjects (median (range); 25^th^ centile; 75^th^ centile).

	Male patients with urethritis-associated STI (Group of cases) (N = 289)	Male patients without urethritis-associated STI (Control group) (N = 192)	P-value ^B^
**Age**	31,3 ^C^ (18,3–50,3); 26,2; 36,2	33,9 ^C^ (19,5–49,7); 29,0; 39,3	<0.001
**First-voided urine volume, ml**	54,5 (6,0–109,6) ^A^; 40,4; 72,0	57,5 (19,0–112,0); 44,0; 74,5	0.076
**Concentration of leucocytes per 1 μl in first-voided urine**	222,6 (0,9–7168,1); 58,2; 497,0	4,6 (0,0–14,9); 2,6; 7,2	<0.001
**Concentration of bacteria per 1 μl in first-voided urine**	29,8 (0,0–20128,0); 13,0; 61,5	4,6 (0,0–19,5); 1,8; 8,5	<0.001
**Total count of leucocytes per whole first-voided urine volume, × 10**^**6**^	10,44 (0,03–546,02) ^A^; 2,92; 23,76	0,27 (0,00–1,14); 0,14; 0,43	<0.001
**Total count of bacteria per whole first-voided urine volume, × 10**^**6**^	1,40 (0,0–58,85) ^A^; 0,76; 2,70	0,25 (0,00–1,82); 0,10; 0,52	<0.001

Median, range, 25^th^ centile and 75^th^ centile are presented in the table because data showed non-parametric statistical distribution.

Only STI monoinfections are displayed, 17 cases with combined STI have been excluded.

^A^ values in group of cases for total count of leucocytes and bacteria per whole first voided urine volume were calculated for 208 patients, excluding 81 patients who had not defined volume of the sample.

^B^ P-value was calculated using Wilcoxon test.

^C^ For age, the mean value is presented instead of the median as for only age parameter the study subjects had normal distribution.

**Fig 3:**
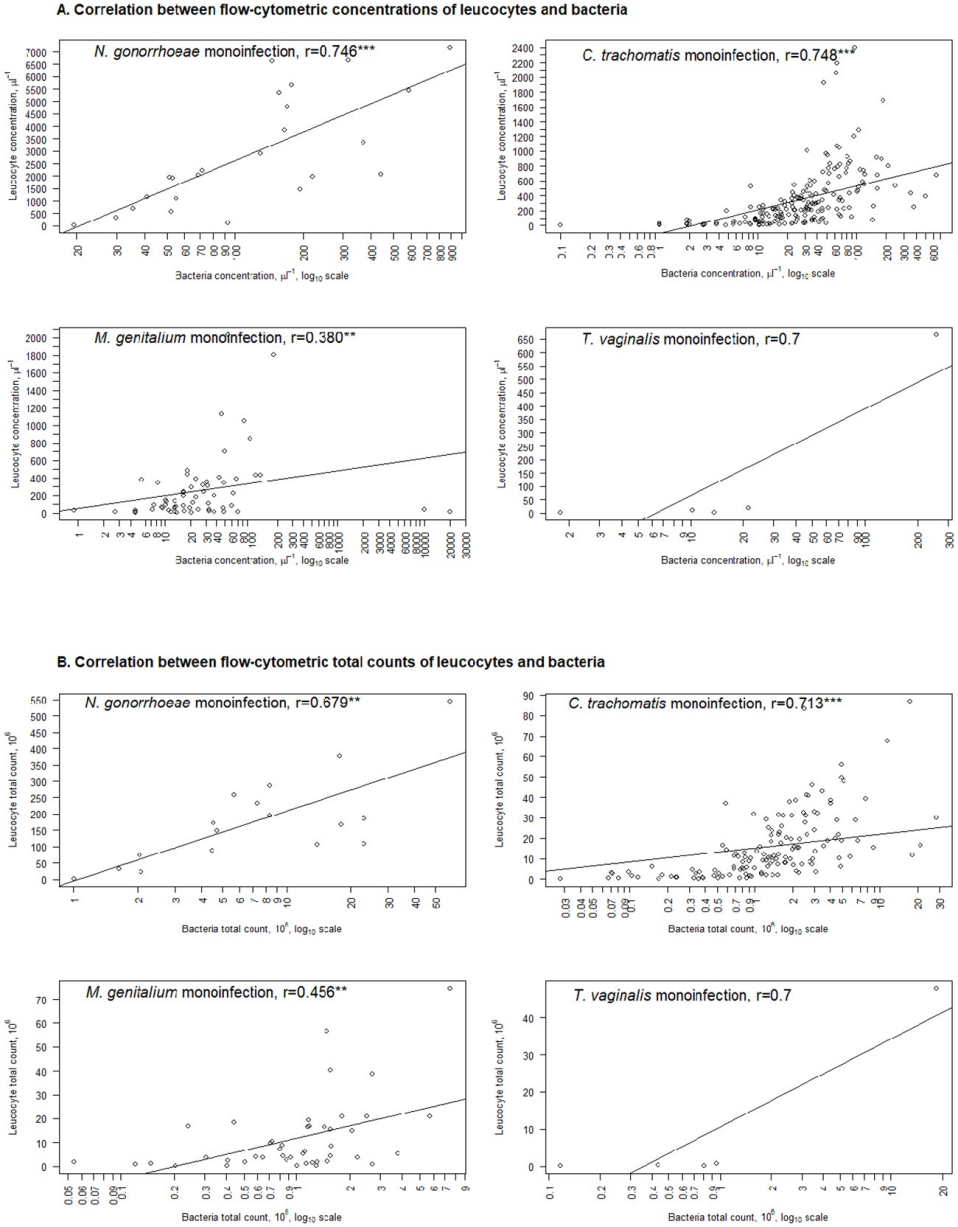
A. Correlation between flow-cytometric concentrations of leucocytes and bacteria in *C*. *trachomatis*, *N*. *gonorrhoeae*, *M*. *genitalium* and *T*. *vaginalis* monoinfection positive cases. B. Correlation between flow-cytometric total counts of leucocytes and bacteria in *C*. *trachomatis*, *N*. *gonorrhoeae*, *T*. *vaginalis* and *M*. *genitalium* monoinfection positive cases. Only STI monoinfections are displayed, 17 cases with combined STI have been excluded. Abbreviations: r—Spearman correlation, *** p < 0.001; ** p < 0.01.

Correlations between the flow-cytometric counts of bacteria and inflammatory reaction in first-voided urine are given in Fig 3. The strongest correlations were revealed in case of CT (r = 0,748 for concentration and r = 0,713 for total count) and NG (0,746 for concentration and r = 0,679 for total count). The weakest correlation between bacteria and inflammatory reaction could be found in MG (r = 0,380 for concentration and r = 0,456 for total count) while it was still statistically significant.

We also tried to check how well our empiric criteria work for the definition of inflammation by flow cytometry in first-voided urine (leucocytes ≥ 15/μl and bacteria ≥ 20/μl) (Table 3). Sensitivity values were over 90% in all cases, except TV. However, if applying such criteria to deciding whether to test for NG, CT, MG and TV, we could miss 21 monoinfections and 2 combined STI cases that makes up 7,5% of all STI positive cases.

**Table 3:** Proportion of urethritis-associated STI positive cases without inflammation in first-voided urine (leucocytes < 15/μl and bacteria < 20/μl) miss rate and sensitivity.

Urethritis-associated STI agent	Cases without inflammation in first-voided urine		False negative rate or miss rate (1-sensitivity)	Sensitivity
	No. of patients	Percentage of all urethritis-associated STI positive cases (N = 306), %		
**Monoinfections and combinations**	23	7.5%	7.5%	92.5%
**Monoinfections only**	21	6.9%	7.3%	92.7%
*Chlamydia trachomatis*	12	3.9%	6.1%	93.9%
*Mycoplasma genitalium*	5	1.6%	7.8%	92.2%
*Neisseria gonorrhoeae*	1	0.3%	4.2%	95.8%
*Trichomonas vaginalis*	3	1.0%	60.0%	40.0%
**Combinations only**	2 *	0.6%	11.8%	88.2%

* These combinations included one CT+NG and one CT+NG+MG.

Abbreviations: CT, *Chlamydia trachomatis*; NG, *Neisseria gonorrhoeae*; MG, *Mycoplasma genitalium*.

Data about the flow-cytometric inflammatory reaction and bacteria in case of combined infections are presented in S2 and S3 Tables. The highest white blood cells (WBC) values were observed in case of combinations with NG (CT + NG and MG + NG), though the latter appeared only in one patient. The CT + NG + MG also appeared in one patient and did not give high WBC counts. The CT + NG combination displayed the highest flow-cytometric counts of bacteria.

## Discussion

We analyzed the profile of sexually transmitted infections among the male patients with STI-associated urethritis consulting an andrologist after high-risk sexual behavior or for prophylactic health and/or fertility check. The most prevalent monoinfection was CT (64.1%), followed by MG (20.9%), NG (7.8%) and TV (1.6%). The total proportion of different combined infections was 5.6%. NG caused the highest inflammatory reaction and the highest bacterial count in first-voided urine, both measured by flow cytometry. CT and MG displayed moderate, and TV showed weak inflammatory reaction in first-voided urine measured by flow cytometry. The strongest correlations between flow-cytometric counts of leukocytes and bacteria were revealed in case of CT and NG.

In this study we used an extensively investigated **control group** for the first time. As a rule, previous studies have described the prevalence of STIs in different populations and/or described the health markers of STI-positive men [12, 15]. We recruited a heterosexual control group without complaints of reproductive tract infections, without signs of urogenital tract inflammation (tested in first-voided urine, mid-stream urine, post-massage urine) and without infectious urethritis (according to PCR test results for CT, NG, TV and MG). This approach gave us the opportunity to compare the flow-cytometric counts and concentration of urine particles (bacteria and white blood cells) in the first-voided urine of men with and without urethritis-associated STIs.

In our previous study [8], we used a **dipstick test** for detection of inflammatory reaction in first-voided urine. However, this test gives only limited quantitative information about an inflammatory reaction in urine, dividing results into five discrete categories. In the current study, we have applied a **flow-cytometry method** that gives continuous quantitative information about an inflammatory reaction in the urine. In addition, the study group was larger in the present study in comparison with our previous study (306 *vs*. 193 STI positive patients). According to Jiang et al. (2011), the results obtained with UF-1000i flow cytometer (Sysmex Medical Electronics Co, Kobe, Japan) for first-morning, mid-stream urine correlated well with visual microscopy examination for WBCs (r = 0.98), and diagnostic performance of UF-1000i was good for WBCs (sensitivity 81.52%) in comparison with visual microscopy examination, but not for bacteria (sensitivity 54.40%). However, visual microscopy examination is not the gold standard for urine bacteria detection. Still, UF-1000i flow cytometer has been found to be more sensitive in predicting the results of visual microscopy examination for leucocytes and bacteria than automated dipstick reflectometer Clinitek Atlas (Bayer Corp, Elkhart, USA) [16]. Boonen et al. (2013) and many other studies using UF-1000i and UF-500i fluorescence flow cytometers have found different cut-off values for concentrations of leukocytes and bacteria for diagnosing urinary tract infections [6, 17]. To the best of our knowledge, there are only a few studies examining performance of UF-1000i/UF-500i fluorescence flow cytometer in diagnosis of urethritis. In a study by Grosso et al. a urethral smear mixed with transport medium was used that is not comparable with our method [18]. In a study by Ito et al. a cut-off point of leukocyte counts of 12.5 WBCs/ml resulted in a sensitivity of 86.9% and specificity of 88.6% for predicting chlamydial infection in asymptomatic men [7]. Men positive for *C*. *trachomatis* (n = 84) had the range 1.8–1666.9 WBCs/μl (median, 43.3 WBCs/μl) in their FVU, which is lower compared with our results (median 222.3 WBCs/μl and range 0.9–2400.2 WBCs/μl). In another study assessing men with symptoms and signs compatible with acute urethritis the inflammatory reaction in FVU assessed by Sysmex UF-1000i flow-cytometer was the following (range and median): 3.9–1918.1 WBCs/μl and 199.0 WBCs/μl for *C*. *trachomatis* (n = 110), 6.9–580.3 WBCs/μl and 185.3 WBCs/μl for *M*. *genitalium* (n = 22) [19]. We found a wider WBC range (1.4–2034.1 WBCs/μl) and a lower median value (108.9 WBCs/μl) for *M*. *genitalium* in our study. There was no information about the bacterial count in the aforementioned studies. Also, the results regarding gonococcal urethritis were not presented in the flow-cytometric study by Ito et al. [19]. Pond et al. [20] have tested this method in MG and CT patients and found its sensitivity slightly lower than in our study. So, our study is the first one to evaluate the performance of flow-cytometry for diagnosis of STI-related urethritis from first-voided urine, while taking into consideration four causative agents and both flow-cytometric bacterial and leucocyte counts. The study gave strong evidence that first-voided urine is a suitable specimen for flow-cytometry analysis. The latter can be considered as a preliminary diagnosing method of urethritis, being a rapid and objective method with considerable sensitivity, devoid of intra-observer and inter-observer variation inherent to microscopic diagnostics, recommended for busy outpatient clinic.

Our study gave some valuable information as concerns practical diagnostics on male STI patients. We succeeded in showing that NG caused the highest **inflammatory reaction** in first-voided urine measured by flow cytometry. This was revealed also in our previous dipstick-based study [8]. CT and MG displayed moderate and TV showed weak inflammatory reaction in first-voided urine measured by flow cytometry. This implies that diagnosing urethritis-associated STIs relying only on flow-cytometric leucocyte and bacteria count in first-voided urine can be misleading as some STI-positive cases may show no inflammatory reaction in first-voided urine. In our study, up 7.5% of all STI-positive cases (23 persons) could have been missed if we had relied on our empiric diagnostic threshold in first-voided urine (leucocytes < 15/μl and bacteria < 20/μl) for flow cytometry. The 2016 European guideline on the management of non-gonococcal urethritis suggests ≥5 polymorphonuclear leukocytes (PMNL) per microscopic high power field (HPF) threshold for Gram-stained smear (GSS) [2]. Geisler et al. found that 6% of chlamydia-infected men had 1 to 4 and 12% had zero PMNL per oil-immersion field while these numbers were 1% and 5% for the men with gonorrhoea [21]. The same ≥5 PMN/HPF threshold was only 63% sensitive and 77% specific for *C*. *trachomatis* infection according to Haddow et al. [22]. In a recent study by Sarier et al., the same threshold value demonstrated 92.9% sensitivity in the diagnosis of gonococcal urethritis (GU) and only 55.6% sensitivity in the diagnosis of non-gonococcal urethritis (NGU), but a threshold value of ≥2 PMNL/HPF reached 100% sensitivity for GU and 92.6% sensitivity for NGU [23]. A study performed by Rietmeijer et al. showed that a significant 2.5-fold increase in chlamydia positivity (from 6.5% to 16.2%) occurred between the 1 and 2 PMNL/HPF strata supporting lowering the diagnostic criteria of the GSS diagnosis of male urethritis [24]. The results of this study were used in Sexually Transmitted Diseases Treatment Guidelines 2015 by Centers for Disease Control and Prevention—the threshold for diagnosing urethritis using GSS was lowered from ≥5 to ≥2 PMNL/HPF [25]. However, a subsequent large-scale study by Moi et al. did not support lowering the cutoff to ≥2 PMNL/HPF [26]. At the same time, a marked degree of both intra-observer and inter-observer variation has been found in the interpretation of the urethral GSS [3, 4], while the sensitivity of this method is also dependent upon the collection technique (the experience of the provider and the device utilized) [12]. Although both methylene blue/gentian violet stain and Gram stain can be used for direct microscopic gonococcal diagnostics, the specificity for *N*. *gonorrhoeae* is not 100%, as other *Neisseria* species may have an identical microscopic appearance [27]. So, the results of our study give adequate sensitivity (92.5%) and miss rate (7.5%) estimates using an empiric diagnostic threshold for urethritis in first-voided urine using flow-cytometry (leucocytes ≥ 15/μl and bacteria ≥ 20/μl).

In addition to WBCs, we also measured the count and concentration of **bacteria** in first-voided urine by flow cytometry. Similarly to WBCs, these parameters were the highest in case of NG. Much lower bacterial counts in the remaining three infections can be explained with the nature of these pathogens. The low bacterial counts and concentrations in case of CT and MG can be related to the fact that CT is an obligatorily intracellular and MG a facultatively intracellular pathogen and flow cytometer cannot detect intracellular bacteria. In addition, both CT and MG are quite small in size. Also, NG can partially multiply inside of WBCs; however, the counts of extracellular NG seem to be high in the urethra in case of acute gonorrhoea. Low bacterial counts and concentrations in case of TV can be explained with the fact that TV is a parasite and its size is comparable with human cells. However, the median concentrations of bacteria were still three to six times higher in the patients with CT, MG and TV than in the men in the control group. According to Dong et al. the microbiomes in male first-catch urine and urethral swab specimens are remarkably similar, independently of STI or urethral inflammation status [28]. It is also important to mention that coronal sulcus and urine have different bacterial communities and coronal sulcus microbiotas are more stable than urine microbiotas [29]. Urethral mucosa always contains normal microbiota and we can speculate that in case of urethral STIs imbalance of this microbiota can occur in the urethra [30]. A similar phenomenon has been described in women in whom STI is frequently accompanied by dysbiotic vaginal microbiota called aerobic vaginitis [31]. The latter is often resolved once the STI is cured. Since dysbiosis of male genital tract microbiota is believed to be significantly related to prostatitis [32–34], we can assume that this may be an important pathogenetic mechanism linking STI and prostatitis.

Although sexually transmitted pathogens infect both males and females, much more is known about the pathogenesis of infections and immune defense of the female genital tract [35–37]. We found different first-voided urine leucocyte and bacteria profiles for particular urethritis-associated STI agents, intentionally excluding combined infections and idiopathic urethritis patients from the group of cases—the differences observed could imply different pathogenic mechanisms of different urethritis-associated STI agents. Other studies also show different clinical and laboratory patterns of particular urethritis-associated STI agents [36, 38, 39]. Also, new scientific methods such as metaproteomic analysis of urine sediments could disentangle the pathogenesis of urethritis [40].

### Limitations of the study

The control group was quite small due to difficulties in performing the studies at the prostate level. The patients in the control group were somewhat older than the patients in the group of cases; however, this difference should not be clinically important. In addition, the number of cases with combined STI and TV monoinfection was too small for drawing any meaningful conclusions. Other possible pathogens associated with urethritis were not analyzed; however, their prevalence is generally low and sometimes the association with urethritis is questionable [35]. Despite the fact that the volume of first-voided urine could not be registered in case of 87 individuals in the group of cases, we believe that this should not distort significantly the information about the total flow-cytometric count of bacteria and leucocytes. We could also observe that the median volume of first-voided urine in both groups was higher than recommended (despite the desired volume of 15–20 ml, the actual volume was 54–58 ml), but this reflects the fact that collecting a smaller volume of urine is difficult for patients. To mitigate effects of this difficulty, we analyzed both concentrations and total counts of WBCs and bacteria and got similar results. As we set up empirical flow-cytometric cut-off values for first-voided urine (inflammatory, if leucocytes ≥ 15/μl and bacteria ≥ 20/μl) to form the control group, we could not assess the specificity and make the analysis of the receiver operating characteristic curve (ROC-curve) for flow-cytometry in diagnosing urethritis-associated STIs. In cases where inflammation and/or bacterial concentration were low in first-voided urine (but the subsequent PCR test confirmed an STI agent) we cannot fully exclude a patient-related (not reported) pre-analytical error in urine collection, e.g. mid-stream urine was collected instead of first-voided urine.

**In conclusion**, CT is the most prevalent urethritis-associated STI among the men consulting an andrologist, followed by MG. A strong inflammatory reaction accompanied by high bacterial concentration in first-voided urine as revealed by flow cytometry is highly predictive of NG infection (sensitivity >95%), while the sensitivity of this method remains slightly lower for CT and MG (>92%) and very low for infrequently occurring TV. This approach can be considered as a rapid and objective screening method in case of suspected urethritis. Further studies are needed to confirm our initial results.

## Supporting information

S1 Table Sysmex UF-500i flow-cytometer. (DOCX)

S2 Table The distribution of inflammatory reaction in first-voided urine according to flow cytometry among patients positive for combined urethritis-associated STI. (DOCX)

S3 Table The distribution of bacteria in first-voided urine according to flow cytometry among patients positive for combined urethritis-associated STI. (DOCX)
