# Supplementary material for: Profile of sexually transmitted infections causing urethritis and a related inflammatory reaction in urine among heterosexual males: A flow-cytometry study
Source: PLoS One. 2020 Dec 2;15(12):e0242227. doi: 10.1371/journal.pone.0242227 (PMC7710099; doi:10.1371/journal.pone.0242227)
Supplement: S1 Table — (DOCX) [file pone.0242227.s001.docx]

**S1 Table. Sysmex UF-500i flow-cytometer.**

| **Principles of work** |
| --- |
| UF-1000i and UF-500i are fluorescence flow cytometers for the quantitative analysis of bacteria, yeast-like cells and leucocytes in human urine, in addition to the analysis of other particles classified out of urine, e.g. erythrocytes and epithelial cells. Two specific fluorescence dyes are used which stain the cells’ nucleic acids and other distinctive cellular parts, thus making it possible to detect bacteria, yeasts and leucocytes with high analytical sensitivity and specificity (1). The tubes with native urine are placed on the sampler of the UF-1000i or UF-500i analyzer directly after opening them. The urine sample will be automatically mixed, and a volume of 1,200 μL will be aspirated for analysis. In the UF-1000i and UF-500i analyzers, the urine sample is split in two separate aliquots and mixed with special reagents at a fixed dilution ratio. The following addition of polymethine dyes will then show optimum staining because specific pH values have been adjusted by the respective diluting reagents.  Polymethine dyes are compounds consisting of x atoms (x = 2n+1; n = 1, 2, 3 …) and x-2 methine groups, and whose x molecule orbitals are occupied in pairs with (x+1) π electrons. These π electrons are completely delocalized and function as highly sensitive photo receptors which absorb the energyrich laser light and subsequently emit fluorescence light (wavelength > 660 nm, near infrared range) when the diluted urine with its stained particles passes the laser beam in the flow cell. Polymethines can be synthetically produced. With the number of methine groups as well as the number and type of branchings, the absorption spectrum of polymethines can be changed and thus easily adjusted precisely to the wavelength of the laser used in the UF instruments.  The urine particles moving through the flow cell pass the laser beam individually and aligned in length, at a high velocity. For each cell, forward and scattered light are simultaneously registered. Optical signals and the characteristics of up to 65,000 particles will be analyzed and are used for the classification of common urinary particles and the generation of additional analytical information (2). |
| **References** |
| 1. Sysmex. UF-500i – High-quality urinalysis at the touch of a button. *The 'second generation' urine Fluoresence Flow Cytometry: the key to modern screening for urinary tract infections. Sysmex Xtra.* [Online] March 2011. [Cited: October 13, 2019.] https://www.sysmex-europe.com/fileadmin/media/f100/Xtra/Xtra_article_The_key_to_modern_screening__of_UTI.pdf  2. —. UF-500i – High-quality urinalysis at the touch of a button. *Sysmex UF-1000i and UF-500i: the modern age of urinalysis. Sysmex Xtra.* [Online] February 2011. [Cited: October 13, 2019.] https://www.sysmex-europe.com/fileadmin/media/f100/Xtra/Xtra_article_UF-Series_The_modern_age_of_urinalysis.pdf |
