# Supplementary material for: Profile of sexually transmitted infections causing urethritis and a related inflammatory reaction in urine among heterosexual males: A flow-cytometry study
Source: PLoS One. 2020 Dec 2;15(12):e0242227. doi: 10.1371/journal.pone.0242227 (PMC7710099; doi:10.1371/journal.pone.0242227)
Supplement: S2 Table — (DOCX) [file pone.0242227.s002.docx]

**S2 Table. The distribution of inflammatory reaction in first-voided urine according to flow cytometry among patients positive for combined urethritis-associated STI.**

| Urethritis-associated STI agent | No of patients ^1^ | Concentration of leucocytes per µl (median (range), 25^th^ centile, 75^th^ centile) | No of patients ^2^ | Total count of leucocytes, × 10^6^ (median (range), 25^th^ centile, 75^th^ centile) |
| --- | --- | --- | --- | --- |
| Control group | 192 | 4,6 (0,0 - 14,9) ^a, b^;  2,6; 7,2 | 192 | 0,27 (0,00 - 1,14) ^c, d^;  0,1; 0,4 |
| **All Combinations** | 17 | 2055,2 (3,6 - 13484,8);  429,2; 4805,6 | 11 | 77,6 (0,3 - 485,5);  32,0; 178,7 |
| CT + NG | 11 | 3418,9 (3,6 - 13484,8) ^a^;  1905,6; 6209,9 | 9 | 149,6 (0,3 - 485,5) ^c^;  75,7; 185,0 |
| CT + MG | 4 | 475,6 (82,7 - 821,4) ^b^;  342,6; 596,8 | 2 | 24,5 (6,1 - 43,0) ^d^;  15,3; 33,8 |
| MG + NG | 1 | 3176,2 | 0 | NA |
| CT + NG + MG | 1 | 11,9 | 0 | NA |

Median, range, 25th centile and 75th centile are presented in the table because data showed non-parametric statistical distribution.

Abbreviations: NA, not available; CT, *Chlamydia trachomatis*; NG, *Neisseria gonorrhoeae*; MG, *Mycoplasma genitalium*; TV, *Trichomonas vaginalis*

^1^ with and without sample volume

^2^ only with sample volume

^a - d^ p<0.017 (Mann-Whitney test with Bonferroni correction for 3 tests [Control *vs.* CT+NG, Control *vs.* CT+MG, CT+MG *vs.* CT+NG])
