# Supplementary material for: Profile of sexually transmitted infections causing urethritis and a related inflammatory reaction in urine among heterosexual males: A flow-cytometry study
Source: PLoS One. 2020 Dec 2;15(12):e0242227. doi: 10.1371/journal.pone.0242227 (PMC7710099; doi:10.1371/journal.pone.0242227)
Supplement: S3 Table — (DOCX) [file pone.0242227.s003.docx]

**S3 Table. The distribution of bacteria in first-voided urine according to flow cytometry among patients positive for combined urethritis-associated STI.**

| Urethritis-associated STI agent | No of patients ^1^ | Concentration of bacteria per µl (median (range), 25^th^ centile, 75^th^ centile) | No of patients ^2^ | Total count of bacteria × 10^6^ (median (range), 25^th^ centile, 75^th^ centile) |
| --- | --- | --- | --- | --- |
| Control group | 192 | 4,6 (0,0 -19,5) ^a, b^;  1,8; 8,5 | 192 | 0,25 (0,0 - 1,82) ^c^;  0,1; 0,5 |
| **All Combinations** | 17 | 110,9 (0,9 - 494,3);  51,2; 212,4 | 11 | 4,6 (0,3 - 25,6);  2,0; 6,6 |
| CT + NG | 11 | 152,8 (3,7 - 494,3) ^a^;  76,4; 220,4 | 9 | 4,7 (0,3 - 25,6) ^c^;  4,2; 8,2 |
| CT + MG | 4 | 107,4 (12,1 - 306,9) ^b^;  41,4; 199,4 | 2 | 1,8 (0,9; - 2,7);  1,3; 2,2 |
| MG + NG | 1 | 103,9 | 0 | NA |
| CT + NG + MG | 1 | 0,9 | 0 | NA |

Median, range, 25th centile and 75th centile are used as descriptive statistics in table because data was with non-parametric statistical distribution.

Abbreviations: NA, not available; CT, *Chlamydia trachomatis*; NG, *Neisseria gonorrhoeae*; MG, *Mycoplasma genitalium*; TV, *Trichomonas vaginalis*

^1^ with and without sample volume

^2^ only with sample volume

^a - c^ p<0.017 (Mann-Whitney test with Bonferroni correction for 3 tests [Control *vs.* CT+NG, Control *vs.* CT+MG, CT+MG *vs.* CT+NG])
